# Integral Fire Protection Analysis of Complex Spatial Steel Structure Based on Optimized Gaussian Transformation Model

**DOI:** 10.1155/2022/6127225

**Published:** 2022-07-21

**Authors:** Zhiyi Wang

**Affiliations:** ^1^State Key Laboratory of Fire Science, University of Science and Technology of China, Hefei, Anhui 230000, China; ^2^School of Energy Engineering, Yulin University, Yulin, Shaanxi 719000, China

## Abstract

The rapid development of complex space steel structure buildings has brought new challenges to structural fire protection analysis and design. On the basis of analyzing the deficiencies of structural fire protection design in existing normative and performance-based design methods and based on the knowledge and research results of fire science, structural engineering, computer simulation, and other disciplines, this paper proposes an improved Gaussian transformation model and applied it to the overall fire performance analysis of this complex space steel structure. The proposed model is based on theoretical research and practical application of the simulation and analysis of the overall fire performance of complex spatial buildings. The coupled analysis model and system integration method of spatial overall structure fire and structure are established. The improved Gaussian transformation model is used to propose the integrity of complex spatial structures. It is a system analysis model for fire analysis. The experimental results show that the designed algorithm model can effectively realize the overall fire performance analysis of complex spatial steel structures and can provide a useful reference for China's building fire protection research, building fire protection design, fire rescue, emergency plan formulation, and other engineering practical applications.

## 1. Introduction

Since the 20th century, the development of spatial structure is very rapid. Different from plane structure, “spatial structure” is regarded as the deepening and expansion of a plane structure, with a three-dimensional structure. Due to the weak tensile performance of concrete used in buildings, steel bars are gradually introduced to improve their tensile performance. Therefore, all kinds of steel have been introduced into buildings, and steel has been used in spatial structures to form the so-called “spatial steel structure.” Moreover, the increase of steel varieties and the improvement of strength make more section steel, steel bars, steel pipes, and cast steel products also applied to spatial structures.

Complex spatial structure refers to building structures with complex structural forms and strong spatial integrity, including various high-rise structures, high-rise, and super high-rise structures, long-span spatial structures, and large underground structures. With the rapid development of the national economy [1], various complex spatial structures emerge one after another. With the convening of large-scale events such as the 2008 Beijing Olympic Games and the 2010 Shanghai World Expo, such structures will develop more rapidly. In addition to bearing the normal load, the engineering structure must also have the ability to resist various accidents and actions, such as fire, explosion, impact, and earthquake to protect the safety of personnel and property in the building. However, these accidental loads and accidental actions have strong uncertainty. Compared with the permanent and variable loads considered in structural design, the action mechanism is much more complex, so it is difficult to be effectively estimated. For a long time, structural designers have ignored the importance of considering structural safety as a whole. After the “9.11” incident, countries all over the world began to recognize and pay attention to the safety of structures in the event of accidents [2].

Due to the increasing scale and complexity of modern space architecture, there are many differences in architectural style, structural form, and use function, as well as new materials. With the development and application of new technologies and new structural forms, the requirements of building fire protection are becoming more and more personalized [3]. The design and research of fire safety in spatial structures encounter many new challenges [4]. At present, the traditional fire safety analysis methods and means cannot meet the design requirements of this kind of new building. Performance-based design is the best way to solve the problem. At present, China has begun to study the performance of large space structures under fire and has made some progress, but there are still many problems [5]. For example, the structural design lacks convenience and easy-to-use system analysis software as a tool used to compare with the current fire prevention code. For the “out of gauge” large-scale buildings, they are mostly solved by expert evaluation. Because this method often depends on personal experience, it is far less scientific than the numerical simulation method. Designers still have difficulties in understanding, learning, and applying performance-based design methods [6]. At present, the existing performance-based design theory in China mainly draws lessons from foreign advanced experience and research results, and there are few original results. In addition, the mechanical analysis and related design methods are mainly focused on the frame structure, and the complex spatial structure has not been involved. One of the main reasons is the lack of appropriate analysis tools and means. Therefore, it is necessary to adopt more advanced and reasonable technical methods for performance-based fire safety analysis [7].

## 2. State of the Art

### 2.1. Systematic Analysis of Fire Safety of Space Integral Structure

Because the interaction of fire site, structure, and personnel in building fire will form a complex system, the fire safety analysis of buildings is a complex system with complex structure, diverse objectives, comprehensive functions, and many factors. And, analyze the complex system process with a strong nonlinear process [8]. A complete analysis process includes fire simulation, structural response analysis, personnel evacuation safety, and property loss assessment. The interaction between fire site and structure is one of the most important contents of fire system analysis. Under the condition of fire, when analyzing the spatial structure, the influence of fire can be regarded as one of many “actions,” which includes many factors such as the temperature field on the component surface. The internal force and deformation of the structure are caused by weakening the material performance and temperature effect. Therefore, it is added to the load action system of the structure as a special load [9]. In fire research, the amount of fuel is usually called fire load. In a broad sense, factors such as space separation and ventilation conditions that significantly affect the speed and degree of fire development can also become fire loads. The spatial separation and ventilation conditions are closely related to the composition and failure state of the structure. Therefore. The firing process in buildings has complex systematic behavior, and the interaction between fire field and structure will form a close coupling relationship. The fire safety analysis of building structures, especially complex spatial structures, should adopt the system method and consider the coupling effect and overall behavior so that the analysis results can reflect this complex systematic behavior [10].

The goal of structural fire resistance design is to prevent the collapse of the whole structure. Therefore, it is more reasonable to use the limit state of bearing capacity based on the overall structure for fire resistance design. Due to the complexity of the problem, there is no fire resistance design code based on the overall performance of the structure [11]. However, structural engineers should still be based on the design goal of the overall fire safety of the structure. From the perspective of overall safety, they should seriously consider using the strength of the structure to carry out effective fire resistance design in the design stage. The fire resistance of the structure is improved by strengthening the integrity of the spatial structure and the number of statically indeterminate times [12]. Instead of relying only on the fire protection of components to achieve the purpose of fire resistance as the traditional design method. Generally speaking [13], the overall fire resistance of the structure can be strengthened in the following ways:

1. Strengthen the continuity and integrity of the structure, such as using connection structure to strengthen the spatial association between components.
2. Improve the statically indeterminate times and crossing capacity. When local failure occurs, the remaining structure can cross the local failure range and form a standby load transfer path, so that the continuous collapse will not continue.
3. Improve the safety reserve of key components. For some important components (such as corner columns or components in the standby load transfer path) or components prone to damage, they are designed to withstand the expected accidental load and have greater safety reserve.
4. Partition isolation strategy, such as the structure composed of multiple independent and integrated substructure systems.
5. Increase the redundancy of the structure. Among them, improving the safety reserve of key components is a very important, practical, and reliable design method for most structures. Its premise is to correctly identify key components in the whole structure. At present, the method of removing components is often used for key component identification and structural integrity analysis [14].

However, the method of removing components is a tentative method, which still has many shortcomings in the application as follows: ① the selection of removed components has a strong human subjectivity and implies artificial assumptions, so there is a risk of error; ② for complex structures, many components often need to be removed, which is blind. There are many assumed working conditions, but it is still difficult to cover all cases, and it is difficult to simulate the combined working conditions. For example, a stadium, namely, Beijing Olympic Stadium uses the demolition method to analyze the overall fire resistance of the structure. Thousands of components have been removed, involving thousands of working conditions, but it is still impossible to consider the integrity; ③ the involvement and dynamic action of component failure cannot be considered; ④ for some structural forms, for example, it is difficult to identify key components such as grid frame and reticulated shell; ⑤ it is still difficult to carry out effective analysis under normal conditions and disaster conditions. In case of fire, the internal force distribution system of the structure will change. Therefore, the key components selected under conventional conditions may not represent the distribution of key components under fire.

Therefore, the component removal method is not suitable for the overall analysis under fire conditions. To quantitatively analyze the overall fire resistance of the structure, in essence, it is necessary that more rigorous and systematic numerical analysis methods should be used for research [15].

**Figure 1:**
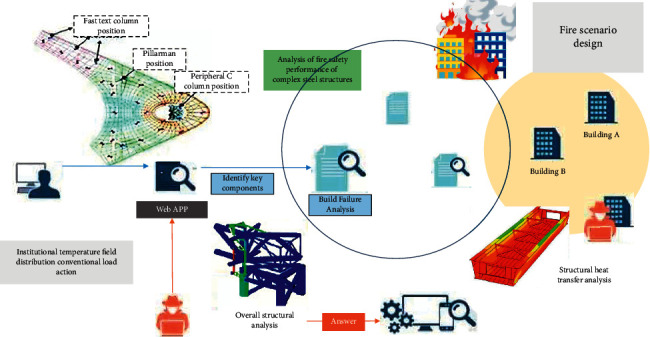
Adopting a systematic approach based on thermal-structural coupling analysis.

Figure 1 shows the overall framework and process for the overall fire protection design and analysis of complex spatial structures using a systematic approach based on thermal-structural coupling analysis. As can be seen from the figure, the fire dynamics model needs to be used first to simulate the fire under the set fire scene. obtained through numerical simulation [16]. The results of the space temperature field under the real” fire is then transferred to the preliminarily designed structural system through the fire-structure coupling model FSCI (fire structures coupled interface) as loads and boundary conditions for structural heat transfer analysis, and the internal temperature field of the structural system is obtained. Then, the overall performance of the structural system under fire is simulated and studied through nonlinear analysis, the change law of its overall mechanical properties and bearing capacity state is found, and the key components or dangerous areas under fire are quantitatively identified. These components or substructures are taken out [17].

According to their temperature environment, loading conditions, and constraints in the space as a whole, the fire resistance analysis of components based on the whole is carried out to verify whether the components fail and further verify whether the whole fails. If failure is found, it is necessary to return to the design stage to readjust the design, including structural design and fire protection design, and then repeat the above process for checking and analysis according to the new design conditions. The system method can be used to analyze the overall fire performance of the structure in any “real” fire scene and the overall fire resistance design of the structure [18]. Both the most unfavorable conditions and the structural response under different fire protection measures (such as sprinkler and ventilation systems) can be considered so that flexible structural fire protection design can be realized, and the design schemes can be compared and optimized. The key technical links in the system method are as follows: (1) how to design the fire scene reasonably; (2) the construction of FSCI is the key link to establish the coupling relationship between fire field simulation and structure analysis; (3) simulation of the whole environment in fire resistance analysis of components [19]. Based on the theoretical knowledge of fire science, structural engineering, computer science, and other fields, the author has carried out preliminary solutions to the above key problems through in-depth and systematic research, which hinders the relatively satisfactory results [20]. These will be detailed in the future. The following is a practical engineering case to illustrate the application of the system method in the fire resistance analysis of the whole structure.

The combination and development of Chinese theoretical research and engineering application make people think that the development of Chinese spatial structure has maintained a good tradition, that is, when people do engineering, they do not carry out it blindly. They must first pass the theoretical and laboratory research and think that the research can be used in practice before it can be applied to engineering. Secondly, the new problems found in the project will also be put forward by putting forward new topics for scholars to study first and then apply to the project.

In recent years, more and more scholars (not only at home but also abroad) have invested a lot of money and time in the research of space steel structures. In 1987, China specially formulated and issued the code for the design and construction of space truss structures to promote the development of steel structures. Secondly, Chinese scholars further studied and improved the nonlinear analysis in the finite element analysis method based on summarizing the existing results, so, they can be applied to the research of spatial components. In short, the development of space steel structures in China is progressing steadily.

## 3. Methodology

### 3.1. Classification of Gaussian Mixture Model

The probability density function of the Gaussian mixture model is defined as follows:

(1) $$\begin{array}{c} p\left(x\right)=\sum_{k=1}^{M}\omega_{k}p_{k}\left(x\right)=\sum_{k=1}^{M}\omega_{k}N\left(x|\mu_{k},\sum_{k}\right), \end{array}$$

where *m* is the mixed number of the model; *ω*_*k*_*p*_*k*_ is the weight factor of the mixed model; and ∑_*k*=1_^*M*^*ω*_*k*_=1; *N*(*x|μ*_*k*_, ∑_*k*_) is the *k*-th single Gaussian probability density function and is

(2) $$\begin{array}{c} N\left(x|\mu ,\sum \right)=\frac{e^{-1/2{\left(x-\mu \right)}^{T}\sum^{-1}\left(x-\mu \right)}}{{\left(2\pi \right)}^{n/2}{\left|\sum \right|}^{1/2}}. \end{array}$$

Estimate reasonable parameters

(3) $$\begin{array}{c} \theta =\left[\omega_{1},\omega_{2},\omega_{3},\cdots ,\omega_{M},\mu_{1},\mu_{2},\mu_{3},\cdots \right.\\ \left.\mu_{M},\sum_{1},\sum_{2},\sum_{3},\cdots ,\sum_{M}\right]. \end{array}$$

The maximum likelihood estimation of the probability density function is maximum, that is,

(4) $$\begin{array}{c} J\left(\theta \right)=\ln\left[\prod_{i=1}^{M}p\left(x_{i}\right)\right]=\sum_{i=1}^{M}\ln\;p\left(x_{i}\right)=\\ \sum_{k=1}^{M}\ln\left[\omega_{k}N\left(x|\mu_{k},\sigma_{k}^{2}\right)\right]. \end{array}$$

To solve the maximum likelihood estimation, the parameter estimation of the Gaussian mixture model is performed by using the maximum expectation (EM) algorithm. The iteration steps of the EM algorithm are.

Step 1: initialize parameters.
(l) Set mean *μ*_1_, *μ*_2_, *μ*_3_, ⋯, *μ*_*M*_ is a random value
(2) Set covariance matrix ∑_1_, ∑_2_, ∑_3_, ⋯, ∑_*M*_ which is the unit matrix
(3) Weighting coefficient of each model set as the prior probability of each model scale, i.e.,
  (5) $$\begin{array}{c} \omega_{i}=\frac{1}{M}, \end{array}$$
where *m* is the number of Gaussian mixed models
Step 2: calculate the prior probability of each component in each Gaussian model
  (6) $$\begin{array}{c} \mathrm{Pr}\left(i|x_{t},\theta^{k}\right)=\frac{\omega_{k}N\left(x_{t}|\mu_{i}^{k},\sum_{i}^{k}\right)}{\sum_{k=1}^{M}\omega_{k}N\left(x_{t}|\mu_{i}^{k},\sum_{i}^{k}\right)}. \end{array}$$
Step 3: update new parameters with prior probability
  (7) $$\begin{array}{c} \omega_{i}^{k+1}=\frac{1}{T}\sum_{t=1}^{T}P_{r}\left(i|x_{t},\theta^{k}\right),\\ \mu_{i}^{k+1}=\frac{\sum_{t=1}^{T}P_{r}\left(i|x_{t},\theta^{k}\right)x_{t}}{\sum_{t=1}^{T}P_{r}\left(i|x_{t},\theta^{k}\right)},\\ \sum_{i}^{k+1}=\frac{\left(\sum_{t=1}^{T}P_{r}\left(i|x_{t},\theta^{k}\right)\left(x_{t}-\mu_{i}^{k+1}\right){\left(x_{t}-\mu_{i}^{k+1}\right)}^{T}\right)}{\sum_{t=1}^{T}P_{r}\left(i|x_{t},\theta^{k}\right)}. \end{array}$$
Step 4: repeat Step 2 and Step 3 until convergence conditions are met
  (8) $$\begin{array}{c} \left|\theta^{t+1}-\theta^{t}\right|<\varepsilon , \end{array}$$
where *θ*^*t*+1^ and *θ*^*t*^ indicates the parameter estimation value of the previous and the previous two times; *E* is the set threshold, usually 10*e* − 5.

### 3.2. Discussion on the Application of New Fireproof Materials in High-Rise Fire Protection System-Specific Application Analysis of Organic Fireproof Materials

In the form and composition of traditional organic fireproof materials, organic fireproof materials are displayed in the form of powder or liquid. If liquid organic fireproof materials need to be used in the application process, the staff also needs to use special solvents to reduce the viscosity of liquid organic fireproof materials and then use stains to make them in advance, to ensure the normal use of the materials. From the perspective of the manufacturing process, the activity of this kind of organic fireproof material is not high. Only when it reaches a high temperature and high pressure can it give better play to specific effects. Therefore, the application scope of traditional organic fireproof materials is relatively narrow, and they are generally used in the production of circuit boards and the use of precision instruments such as electrical components. The new organic fireproof material fully makes up for the shortcomings of traditional fireproof materials. It no longer needs solvent to reduce viscosity and impregnation process in the application process. It can be used normally under normal temperature and pressure, and its application range is greatly increased. In the current application, this new type of organic material will not produce smoke and toxic gas in case of fire, which provides good environmental support for people's escape. In the current fire protection system of high-rise buildings, this kind of material is favored by people and comprehensively improves the life safety of people living in buildings.

### 3.3. Application Analysis of Sprayed Fireproof Materials

At present, sprayed fireproof materials are not widely used in high-rise buildings in China. This material is mainly used in tunnel engineering and relevant parts of industrial equipment. Sprayed fireproof materials can be well integrated with water, especially cement-sprayed fireproof materials will turn into mud after encountering water and then sprayed onto the wall to be protected through air compression.

### 3.4. Application Analysis of Calcium Silicate New Fireproof Material

In the current fire protection system of high-rise buildings, calcium silicate fireproof material is the most widely used. This material takes into account the characteristics of applicability and economy. It is one of the most frequently used fireproof materials in high-rise buildings. In the current application, the most advantageous application of calcium silicate fireproof material is to construct the air duct, to have a good fire prevention effect.

## 4. Result Analysis and Discussion

### 4.1. Analysis of Engineering Examples

The project is a panoramic painting gallery of radio and television transmitting towers in a province. The TV Tower is 388 inches high, and is divided into four parts: tower base, tower body, tower, and mast. The tower includes the underground *l* floor, the upper four floors, and the roof. The tower body is circular from 24.6 m to 213.8 m in elevation. The steel structure is of Q345 type. The panoramic gallery is a very special indoor exhibition hall in the building, which is distributed on the third and fourth floors of the tower and is a large space with a height of 22 m on the second floor. Its plane area reaches nearly 2000 m: oil painting is distributed around the whole space. The exhibition hall of the oil painting adopts 4 T linen as canvas and 6 *t* mineral powder oil painting paint. Canvas and pigment are combustible, with a large fire load and rapid fire spread. The canvas is close to the surrounding supporting steel structure system, which is very dangerous to fire. In case of fire, the nearby steel structure will be exposed to the radiation of the fire source directly. The top beam system will also be affected by high-temperature flue gas, which makes the whole structure system in an uneven temperature field. In addition, under the joint action of high temperature and original load, the stress condition inside the structure system will change greatly, and the internal force redistribution will appear in the whole structure system. In this case, whether the original design can meet the requirements of bearing capacity and function needs to be analyzed in depth to make clear. This paper uses the system analysis method of the overall fire resistance of the structure system, and systematically simulates and analyzes the fire scene and the fire safety performance of the most dangerous panoramic painting hall in the whole building under the setting fire. The fire safety performance of the painting hall is analyzed.

### 4.2. Fire Scene Design and Simulation

Fire scene simulation is to predict the temperature change and smoke movement in the building in the future according to the fire partition type, fire load type, quantity and distribution, building opening, and active fire fighting facilities. Temperature is an important factor to influence the fire resistance design of the structure. Therefore, fire scene simulation is not only the basis of performance-based fire safety design but also the basis of fire resistance design. In the project, according to the risk analysis, there are many fire scenes designed. This paper selects one of them to explain the analysis process. The scene is designed to fire the canvas. The ignition point is set at the bottom of the canvas near the outer steel column. According to the analysis of the structure of the painting gallery, the fire in this place has the greatest influence on the surrounding support and roof structure system. Because of the large space of the panoramic gallery, according to the number of combustible materials in the gallery, the canvas is flammable and fast to burn. Referring to the similar characteristic fire load survey data at home and abroad, it is determined that the fire type of this simulation is super-fast T2 fire and the scale is determined as 10 MW. Since the most unfavorable conditions are considered, air supplement and smoke exhaust are not considered, and spray failure is assumed.

According to the design of the fire scene, bfiresasn 43 (an integrated fire structure coupled analysis system developed by the author) is used for fire simulation analysis, and the result data of surface ambient temperature, heat flow, and radiation of each component are obtained.

**Figure 2:**
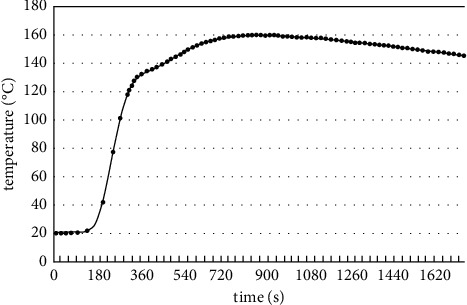
Steel structure time-temperature curve diagram.

Through calculation and simulation, the steel structure time-temperature curve diagram can be seen in Figure 2. The maximum temperature peak appears at about 210 s. It reached 896°C and then began to decline rapidly. It can be seen from the distribution of the temperature cloud diagram that the highest temperature occurs near the roof beam system directly above the fire source, and the components here are in a high-temperature environment, so they are very dangerous. From the distribution of the temperature field, it can be seen that, in the large space structure, the space temperature field is a physical field that changes dynamically with time and space. Any point in the space is affected by the radiation and convection of the fire source and high-temperature flue gas at a certain time to produce different ambient temperatures. Therefore, this time-space varying temperature field needs to be accurately applied to the overall structure as a load condition.

### 4.3. Structural Thermal Analysis

**Figure 3:**
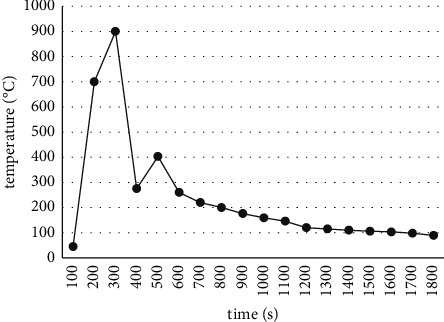
Space-ambient temperature curve.

Through the FSCI (fire structure coupled interface) model program of bfiresasn51 (an integrated fire structure coupled analysis system developed by the author), the spatial dynamic temperature field *t* (*x*, *y*, *Z*, *I*) obtained by fire simulation is applied to the whole structure for the transient analysis of the structural system, as can be seen in Figure 3. Through the state thermal analysis, the changes in the internal temperature field of the two structural systems are obtained.

**Figure 4:**
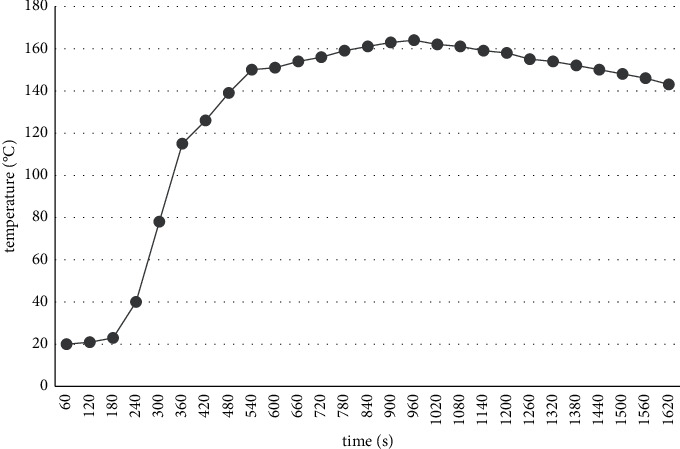
Temperature-time curve of steel structure.

Figure 4 shows the temperature variation of the steel structure member above the fire source with time. As can be seen from the figure, due to the thermal effects such as radiation and convection, the internal temperature of the steel structure reaches a peak at about 930 s, and the maximum temperature is about 160°C, which occurs in the steel beam above the fire source.

### 4.4. Fire Resistance Analysis of the Whole Structure

The traditional fire resistance design method based on tests cannot well consider the actual stress and restraint of components, nor can it consider the middle structure of the whole structure.

For the factors such as the interaction force between components and the change of load transfer path, the overall fire resistance analysis method based on the structural system can be used in the design.

The above problems shall be comprehensively considered in the plan. In this project, the general finite element software analyst is used to analyze the overall structure of the panoramic painting Museum. The type of steel is Q345. The parameters of steel at high temperatures, such as specific heat, thermal conductivity, and expansion coefficient, are taken according to the European code, the density is 7850 kg/m^3^, and the stress-strain relationship adopts the model in Eurocode 3. The element simulating beam and column is beam 44 element.

The thermal parameters and structural load of steel under fire shall be carried out as per the technical code for fire protection of steel structures in buildings (cecs200:2006) and load code for building structures (GB50009-2001), and the load effect combination is as follows:

(9) $$\begin{array}{c} q=1.0G_{k}+0.7Q_{k}+1.0\Delta T, \end{array}$$

where *G* is the standard value of permanent load; QK is the standard value of live load; and △*t* is the temperature change of the component (considering the temperature effect).

**Figure 5:**
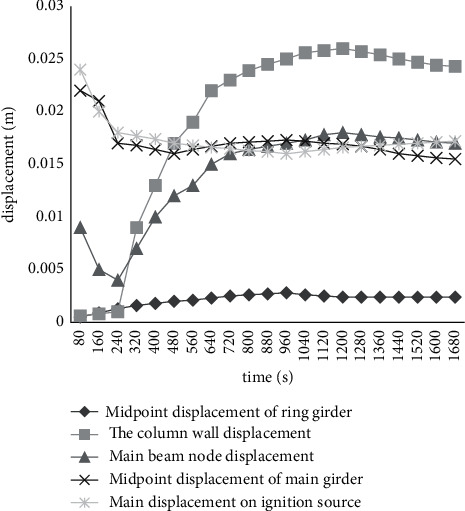
Position-time curve of beams and columns near the fire source.

**Figure 6:**
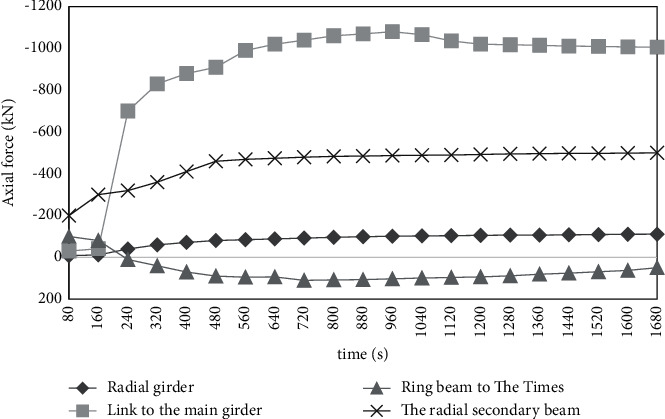
Axial force diagram of the steel structure system.

Figures 5 and 6 show the mechanical response of the structural system of the gallery under the action of simulated fire.

Under the action of fire, the displacement of the top of the peripheral column at the most unfavorable position is 23 mm. The maximum stress in the column is 342 MPa. It indicates that the member may have yield failure. It cannot meet the requirements of bearing capacity. As can be seen from Figure 6, under the action of high temperature in fire, due to the expansion of the beam, it will produce a large horizontal thrust on the surrounding peripheral support columns, coupled with the action of vertical load. A large additional bending moment is generated in the column. Thus, a high-stress level is generated in the column. Without fire protection, the steel structure column will yield, thus losing the bearing capacity. For example, in column w-l, under the joint action of high temperature and beam load, its axial force and stress are quite large.

### 4.5. Fire Resistance Analysis of Components

The coupling model is used to analyze the overall fire resistance of the structural system under fire, which can examine the overall mechanical properties of the structural system from a macroperspective, study the force transmission path of the structure under fire, and quantitatively identify the dangerous areas and key components of the structure. However, the overall analysis is carried out on a large scale. According to the needs of the analysis, the model and parameters will be simplified, and the design should be implemented on the components. Therefore, it is also necessary to conduct a more in-depth detailed analysis of key components based on the results of the overall analysis to obtain more accurate local analysis results. From the results of the overall analysis, it can be seen that the steel columns and beams near the fire source are greatly affected by the fire. As the main load-bearing components, they have high risk. In this paper, taking the radial main beam as an example, the fire resistance analysis and bearing capacity checking calculation of key components are carried out. As a component in the overall structure, when analyzing the fire resistance performance of the main beam, we must consider its state in the overall environment, simulate the surrounding environment and the effect of the component on it, and make the ambient temperature, constraint, and stress state of the component consistent with that in the overall structure.

**Figure 7:**
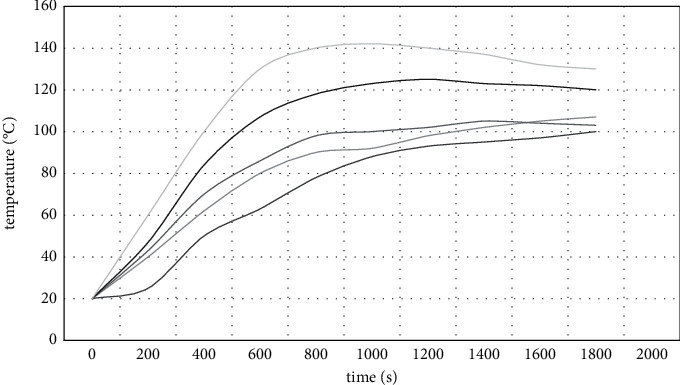
Curves of temperature versus time at different interfaces on the beam.

Firstly, ANSYS is still used to study the fire resistance of a single component. In the analysis, the solidt0 element is used to establish the main beam model, and the thermal structural coupling analysis is carried out.  Figure 7 shows the internal temperature field of steel beam obtained by transient thermal analysis of components in a real fire environment.

Figure 7 shows the temperature change curve of different parts of the main beam with time. It can be found from the figure that the highest temperature occurs near the connection between the beam end and the column, reaching a peak of 146°C at 9308. It can be seen from the results. Because the analysis of a single component is carried out in the overall environment, the ambient temperature and stress state around the component are consistent with those in the overall structure. Therefore, the analysis results are not much different from those in the overall analysis. This also shows that although some simplification has been made in the overall analysis, it can still meet the accuracy required by the project as long as it is handled properly. Since the “real” fire scene is used to simulate the temperature rise curve, the thermal analysis of the structure is carried out. The temperature in the beam section is different from that in the length direction and changes dynamically with time. Many previous studies usually assumed that the internal temperature of components was uniform, which was inconsistent with the actual fire.

After obtaining the temperature field distribution results in the beam. Structural stress analysis will be carried out. The load on the steel beam is consistent with the actual situation. Two working conditions are considered for end restraint: ① similar to the overall analysis, considering the situation of no protection, the restraint effect of the column on the beam simulates the actual situation and adopts a semirigid connection; ② due to the importance of the column, considering that the column is well protected from fire, it has large lateral stiffness. At this time, the beam-column can be assumed to be a rigid connection.  Table 1 summarizes the deformation and stress of the main beam under the two working conditions. We can see from the table that the connection between the beam end and the other components has higher stress levels and deformation than in the middle of the beam. The reception of the beam end plastic zone occurred earlier with the highest probability of fracture. The reason is that the main beam is restrained in expansion deformation due to the interaction with other surrounding components, and the components at the beam end connection are extruded and deformed, resulting in high stress, causing local compression buckling failure at the end.

**Figure 8:**
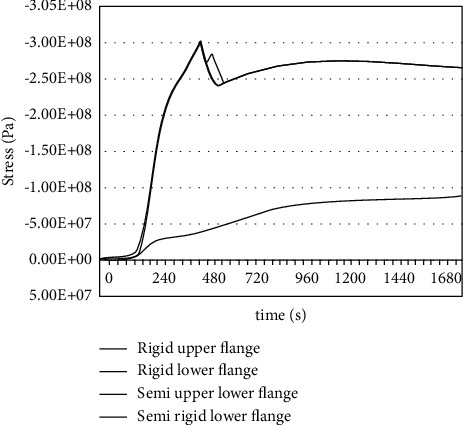
Comparison of beam end stress change under two working conditions.

Figure 8 shows the changes in beam end stress under two working conditions. As can be seen from Figure 8, the stress at the beam end in the case of a semirigid connection is smaller than that in the case of a rigid connection, which is beneficial to improving the fire resistance of steel beams. However, it will produce a large lateral thrust on the column, which is easy to cause the additional stress of the column to be too high and cause instability and failure. If the column is well protected against fire, the beam end will make the main beam more prone to failure due to higher axial and torsional constraints. This contradiction reflects the advantages of the overall analysis, which requires designers to analyze as a whole.

To coordinate the contradictory relationship between components. It can also be seen from the analysis that the members are under the joint action of high temperature and load. The plastic area appears first at the beam end, which is the dangerous section of the main beam. This is different from the conclusion that the midspan is the dangerous section in the previous experimental research. This is mainly because the actual constraints of the members are considered in the fire resistance analysis of the members based on the whole.

**Figure 9:**
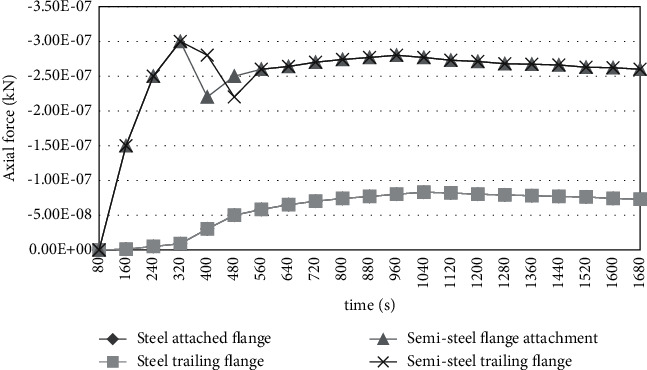
Stress diagram at different positions.

Through the finite element analysis of key components in Figure 9, we can see that when the ambient temperature reaches 895.57°C, the internal stress of the beam exceeds its bearing capacity limit, resulting in damage to the main beam. In the state of no protective layer, the main beam does not meet the fire resistance design requirements, so we first consider adopting fire protection.

Paint for fire protection: according to the overall analysis data, the radial main beam located on the outermost ring of the second floor near the fire source is the place with the highest temperature in the fire, the maximum stress in the main beam is 286 MPa, and the maximum ambient temperature is about 895.57°C. The beam is an I-shaped steel beam, with a flange width of 0.3 m, web thickness of 0.012 M, flange thickness of 0.016 m, web length of 0.8 m, steel grade Q345, section area *a* = 188.16 cm^2^, and strong shaft section modulus of *W* = 4820 cm^3^. The axial binding force at both ends is 1340920 n. The bending moment at the beam end is 1604890 nm and 470382 nm, and the wind = fit = 1.0, and the heat conduction coefficient of the fireproof protective layer material is 9 = 0.10 w/(m°C), respectively. Now, the thickness of the fire protection layer is designed for the components: set the thickness of the protection layer as DL, and calculate the section strength load ratio *R*.

(10) $$\begin{array}{c} R=\frac{1}{f}\left(\frac{N}{A_{n}}+\frac{M_{x}}{\gamma_{x}\omega_{ux}}\right)=\frac{1}{275}\left(\frac{-1340920}{18816}+\frac{1604890000-470382000}{1.05\times 4820\times 10^{3}}\right)=0.556,\\ T_{d0}=579.84^{{}^{\circ}}\text{C},\\ N_{\text{EX}}^{\prime }=\frac{\pi^{2}EA}{1.1\lambda_{x}^{2}}=\frac{\pi^{2}\times 2.1\times 10^{5}\times 18816}{1.1\times 22^{2}}=7.32\times 10^{7}N,\\ R_{x}=\frac{1}{f}\left[\frac{N}{\varphi_{x}A_{n}}+\frac{\beta_{mx}M_{x}}{\gamma_{x}\omega_{x}\left(1-0.8N/N_{EX}^{\prime }\right)}\right]=0.518,\\ e_{1}=\frac{\beta_{mx}M_{x}}{\gamma_{x}W_{x}\left(1-0.8N/N_{EX}^{\prime }\right)}\cdot \frac{\varphi_{x}A}{N}=-2.9,\\ e_{2}=\frac{\eta \beta_{ty}M_{y}}{\varphi_{by}^{\prime }W_{y}}\cdot \frac{\varphi_{x}A}{N}=0,\\ \varphi_{by}^{\prime }=1.07-\frac{\lambda_{y}^{2}}{44000}\cdot \frac{f}{235}=0.73,\\ R_{x}=\frac{1}{f}\left(\frac{N}{\varphi_{y}A}+\eta \frac{\beta_{tx}M_{x}}{\varphi_{by}^{\prime }W_{x}}\right)=0.561,\\ R_{x}=\frac{1}{f}\left(\frac{N}{\varphi_{y}A}+\eta \frac{\beta_{tx}M_{x}}{\varphi_{by}^{\prime }W_{x}}\right)=0.561,\\ e_{1}=\frac{\beta_{mx}M_{y}}{\gamma_{y}W_{y}\left(1-0.8N/N_{EY}^{\prime }\right)}\cdot \frac{\varphi_{y}A}{N}=0,\\ e_{2}=\frac{\eta \beta_{tx}M_{x}}{\varphi_{bx}^{\prime }W_{x}}\cdot \frac{\varphi_{x}A}{N}=-1.92,\\ d_{i}=5\times 10^{-5}\times \frac{\lambda_{i}}{{\left(T_{d}-20/t+0.2\right)}^{2}-0.044}\cdot \frac{F_{i}}{V}=0.035\text{ m}. \end{array}$$

Therefore, when the thickness of the fire protection layer of the steel beam is at least 35, and the steel beam can meet the fire resistance requirements of bearing capacity and stability. However, the number and surface area of steel beams in the project is huge. Painting such a thick fireproof coating will cost a lot of money. Therefore, more economical and reliable means can be used for fire protection under the guidance of overall system analysis. Under the condition of reducing the thickness of fire-retardant coating, the structural design of the beam-column connection is modified, and the beam-column connection is changed into sliding support so that the structural system can not only meet the original design requirements but also meet the fire resistance of beam-column. It also saves the use of fire-retardant coating, which reflects the advantages of performance-based design. In addition. The higher stress of the steel column in the core tube is mainly due to the redistribution of internal force due to the overall effect of the structural system and is caused by the temperature effect caused by its internal high temperature. The fire resistance strength cannot be improved by relying on the fire-retardant coating, but the internal force should be reduced by relying on the overall structural coordination.

## 5. Conclusion

The rapid development of China's economic construction not only makes the development of space steel structure more important but also provides opportunities and challenges for the development of steel structure. In recent years, the holding of various grand events in China has provided conditions for the spatial steel structure, which makes China have more opportunities and challenges, and the required forms are also diverse. The external forms respect the originality and applicability and put forward higher requirements for the architectural structure design.

Nowadays, from the development of space steel structure, the development in the next few years requires the span of space steel structure to be larger and larger, and the size of reticulated shell structure is also gradually increasing, which needs more technical support. At the same time, the demand for architectural form is also changeable, which urges structural engineers to achieve structural innovation in structural design. On the whole, the development of space steel structure is still very optimistic. There is more room for development in the future, and more people are needed to participate.

Structural fire protection design is by no means an isolated process. Studying the mechanical properties and fire protection design methods of the whole structure under real fire simulation is an important step in the development of structural engineering and fire safety engineering. This paper summarizes the shortcomings and disadvantages existing in the current fire resistance research and design of steel structures and integrates the knowledge and research results of many disciplines, such as fire science, structural engineering, and computer simulation. Based on the theoretical research and practical application of the simulation and analysis of the overall fire performance of complex space buildings, the fire structure coupling analysis model and system integration method of the spatial integral structure are established. The system analysis model of integrated fire protection analysis of complex spatial structures is proposed, and an example is studied.

Through systematic research, this paper draws the following conclusions with reference to relevant research and design work:

1. Fire environment is the basic condition for structural fire resistance analysis. The space temperature in the real fire simulation is the space field form *t* (*x*), *Z*_*o*_, that changes with time, showing uneven changes in the horizontal and vertical directions. This heterogeneity has a great impact on the stress state of the space steel structure and causes changes in the internal force and equilibrium state of the structure system. Therefore, it is not suitable for large space structures to use ISO curve and space uniform temperature rise assumption as the ambient temperature condition of fire resistance.
2. The structural system often shows strong integrity under the action of fire. The internal force redistribution of the structural system caused by uneven temperature field will make overall fire resistance enhanced. It is found that some forms of spatial structure system take the lead in structural damage away from the fire source.
3. The mechanical properties of components in the overall environment are often very different from those based on the test, so in the fire resistance design of components,
4. the influence of the overall effect should be fully considered, and the design method in the code should be flexibly used to design more practical components.
5. Based on the overall analysis of the system, in line with the principles of safety, effectiveness, and economy, a variety of technical means can be flexibly used to strengthen the fire performance of the structure, rather than relying solely on fire retardant coatings, to truly reflect the essential connotation of performance-based design.

China should speed up the research pace of overall fire protection analysis and supporting design methods of steel structures and give full play to the advantages of performance-based design. In the future,

the development of performance-based design technology for steel structures should be promoted from the following aspects:

1. Strengthen the research on fire scene design methodology, ensure the comprehensiveness, objectivity, and systematicness of fire scene design, and put forward suggestions for engineering practice standardized fire scene design method.
2. Indepth study of the overall mechanical properties of steel structure buildings with various structural forms under fire, to lay a theoretical foundation for the performance-based analysis of steel structure basics. At the same time, we should actively expand the application of numerical simulation and computer technology in fire protection.
3. Enrich and improve the basic research data of fire and structural fire resistance performance, provide accurate performance-based indicators, and accumulate a basis for performance-based application basic data.
4. The combination of numerical simulation and experimental research is adopted to improve and deepen the research on the systematic design method based on the whole. Strengthen the cooperation between scientific research institutions and front-line units, gradually apply the new technology to practice, and constantly test, improve, and develop the new technology in practical application. So that it can truly achieve the purpose of engineering practicality.
5. Actively strengthen and improve the professional level of performance-based fire protection of architectural designers and fire management personnel, so that they can understand, accept, and be familiar with the integrated fire protection design method.

## Figures and Tables

**Table 1:** Deformation and stress of the main beams under the first and second operation.

Working condition	Presplit *X Y*	Cracked *X Y*	Crack *X Y*	Destroy *X Y*
Load, kN	0∼160	0∼220	0∼200	200∼420
Loading moment, 0 kN**·**m^−1^	3081	4671	3876	8110

## Data Availability

The labeled dataset used to support the findings of this study is available from the corresponding author upon request.
